# Density Distribution of Strongly Quantum Degenerate Fermi Systems Simulated by Fictitious Identical Particle Thermodynamics

**DOI:** 10.3390/e27050458

**Published:** 2025-04-24

**Authors:** Bo Yang, Hongsheng Yu, Shujuan Liu, Fengzheng Zhu

**Affiliations:** 1 Center for Fundamental Physics, Hubei Polytechnic University, Huangshi 435003, China; yuhongsheng@hbpu.edu.cn (H.Y.); liushujuan@hbpu.edu.cn (S.L.); fzzhu@hbpu.edu.cn (F.Z.); 2 School of Mathematics and Physics, Hubei Polytechnic University, Huangshi 435003, China

**Keywords:** fermion sign problem, path integral molecular dynamics, interacting fermionic system, entropy

## Abstract

The exchange antisymmetry of identical fermions leads to an exponential computational bottleneck in ab initio simulations, known as the fermion sign problem. The thermodynamic approach of fictitious identical particles (Y. Xiong and H. Xiong, J. Chem. Phys. 157, 094112 (2022)) provides an efficient and accurate means to simulate some fermionic systems by overcoming the fermion sign problem. This method has been significantly promoted and used by National Ignition Facilities for the ab initio simulations and is believed to have wide application prospects in warm dense quantum matter (T. Dornheim et al., arXiv: 2402.19113 (2023)). By utilizing the fictitious identical particles in the bosonic regime and constant energy extrapolation method (Y. Xiong and H. Xiong, Phys. Rev. E 107, 055308 (2023); T. Morresi and G. Garberoglio, Phys. Rev. B 111, 014521 (2025)), there are promising results in simulating the energy of strongly quantum degenerate fermionic systems. The previous works mainly concern the energy of Fermi systems or only consider situations of weak quantum degeneracy. In this study, we extend the concept of the constant energy extrapolation method and demonstrate the potential of the constant density extrapolation method to accurately simulate the density distribution of fermionic systems in strongly quantum degenerate conditions. Furthermore, based on the energy derived from the constant energy extrapolation method, we present simulation results for the entropy of fermions.

## 1. Introduction

The exact numerical simulation of fermionic quantum many-body systems has always been a research focus in quantum systems such as condensed matter and warm dense matter. Many unresolved issues rely on the exact numerical simulation of fermionic quantum many-body systems, such as the Fermi–Hubbard model involved in high-temperature superconductivity and warm dense quantum matter in inertial confinement fusion. Unfortunately, the exact numerical simulation of fermionic quantum many-body systems is often severely constrained by the fermion sign problem [1,2,3,4,5]. Despite the applicability of Path Integral Monte Carlo (PIMC) [6,7,8,9,10,11,12,13,14,15] and Path Integral Molecular Dynamics (PIMD) methods [16,17,18,19,20,21,22,23] to bosonic systems with particle numbers exceeding $10^{3}$, the exact numerical simulation of fermionic systems has long been limited to relatively small quantum systems due to the presence of the fermion sign problem. About 30 years ago, Ceperley et al. devised a fixed node method [24] to circumvent the fermion sign problem, but this method relies on the nodal properties of the fermionic wave function and may introduce uncontrollable errors in cases where the nodal properties of the wave function are not known in advance [25]. In recent years, methods such as the blocking permutation method, Configuration Path Integral Monte Carlo (CPIMC), and diagrammatic Monte Carlo [24,25,26,27,28,29,30,31,32,33,34,35] have also been applied to simulate fermionic quantum many-body systems. However, these methods are still limited by conditions such as the temperature and the number of fermions that can be simulated.

In 2022, Xiong and Xiong [36] proposed the concept of fictitious identical particles to overcome the fermion sign problem and perform ab initio numerical simulations of fermionic systems. This method holds the promise of extending the numerical simulation of fermionic quantum many-body systems to extremely low temperatures [37] and large-scale fermionic systems with particle numbers comparable to bosonic systems. The main idea of this method is to introduce a real parameter ξ that can continuously vary for the fictitious identical particles (where ξ=1 represents bosons and ξ=−1 represents fermions), and then use the exact numerical simulation results in the bosonic region ξ≥0 to extrapolate the properties of the fermionic system. By performing numerical simulations of quantum systems within the ξ≥0 interval (where there is no fermion sign problem) and using the isothermal extrapolation method [36], Xiong and Xiong accurately calculated the energy of fermionic systems at moderate and high temperatures with significant fermionic statistical effects present.

Recently, Dornheim et al. [38] confirmed the value of the isothermal ξ-extrapolation method based on fictitious identical particles [36] under warm dense matter conditions. In particular, Dornheim et al. [38,39,40,41,42] systematically generalized the isothermal ξ-extrapolation method based on fictitious identical particles, efficiently and accurately simulating important physical quantities such as density distributions, static structure factors, and static linear density response functions. In a groundbreaking paper, Dornheim et al. [39] made significant progress in warm dense matter conditions using PIMC, surpassing previous best results for tens of fermions by successfully, precisely simulating one thousand electrons. This laid a solid methodological foundation for a wide range of quantum systems, particularly warm dense matter relevant to inertial confinement fusion. Most recently, experiments conducted at the National Ignition Facility [40] excitingly discovered new experimental data that remarkably align with the new PIMC simulation results based on the ξ-extrapolation method. Subsequently, in another groundbreaking paper, Dornheim et al. [41] demonstrated that the ξ-extrapolation method within PIMC provides powerful tools for solid hydrogen and strongly compressed beryllium. They found that for warm dense hydrogen, employing direct PIMC requires a massive computation of $O(10^{7})$ CPU hours, yielding results entirely consistent with the efficient ξ-extrapolation method [42]. Furthermore, they used the ξ-extrapolation method to address previously perplexing finite-size effects in scenarios involving a large number of fermions.

It is important to emphasize that the isothermal extrapolation method based on fictitious identical particles is not applicable to systems at low temperatures or in highly quantum degenerate situations [37,38]. At low temperatures, within the range ξ≥0, the energies of fictitious identical particles cluster around a single energy value (referred to as the “black region”, where the energies of particles with different temperatures and ξ are almost the same, in the paper [37]), while the energies of fermionic systems are significantly different. Therefore, it is not possible to obtain information about ξ=−1 from data within ξ≥0 by the isothermal extrapolation method. It can also be proven that at zero temperature, in the 0≤ξ≤1 bosonic region, the ground-state energy is independent of ξ [43]. Xiong and Xiong discovered that by utilizing the method of constant energy semi-extrapolation [37], it is possible to bypass the “black region” and potentially accurately infer the energies of some fermionic systems at extremely low temperatures (even at the ground state). With the constant energy semi-extrapolation method, it is easy to verify that highly accurate energy results from zero temperature to high temperature can be obtained for hundreds of non-interacting fermions in a harmonic trap. It is worth noting that the constant energy semi-extrapolation method proposed by Xiong and Xiong has partially overcome the challenges of simulating energy in highly quantum degenerate scenarios. In a recent breakthrough by T. Morresi and G. Garberoglio [15], a tailored extrapolation scheme is proposed based on parametrized partition function of fictitious identical particles, and the simulation of energy is shown to be in good agreement with experimental results for normal liquid ^3^He.

However, an outstanding question remains: if the constant energy semi-extrapolation can accurately simulate the energy of a certain fermionic system in a highly quantum degenerate state, can fictitious identical particles accurately simulate physical quantities such as density distributions? Density distributions are important in the study of liquid Helium systems and condensed matters. This also lays the foundation for studying other properties of Fermi systems. The main purpose of this paper is to address this question and provide a method for simulating the density distribution of fermionic systems in highly quantum degenerate states.

More specifically, expanding the concept of constant energy semi-extrapolation, in this paper, we will develop the method of constant density semi-extrapolation to simulate the density distribution of fermionic systems. In comparison to constant energy semi-extrapolation, the data regarding the density distribution in the bosonic region is somewhat more complex, but the basic approach remains similarly straightforward. Our research indicates that the constant density semi-extrapolation method holds promise for accurately numerically simulating the density distribution of some highly quantum degenerate fermionic systems.

The structure of this paper is as follows: In Section 2, we first provide a brief introduction to some fundamental knowledge in ab initio simulations with PIMD and elucidate how to use appropriate ξ-extrapolation methods to study the density distribution of fermionic systems. In Section 3, we present the energy, density distribution, and entropy of fermionic systems in a two-dimensional harmonic trap obtained using different ξ-extrapolation methods. In Section 4, we provide a brief summary and discussion.

## 2. Fictitious Identical Particle PIMD and Constant Density Semi-Extrapolation Method

### 2.1. Fictitious Identical Particle PIMD

For *N* spin-polarized interacting particles, the Hamiltonian operator is

(1) $$\hat{H}=\frac{1}{2m}\sum_{l=1}^{N}{\hat{p}}_{l}^{2}+V\left(r_{1},\cdots ,r_{N}\right),$$

where ${\hat{p}}_{l}$ represents the momentum of each particle, and $V\left(r_{1},\cdots ,r_{N}\right)$ denotes the interactions between particles and harmonic trap potential. For fictitious identical particles, the parametrized partition function takes the following form [15,36,37,38,39,40,41,42,43]:

(2) $$Z\left(\beta ,\xi \right)∼\sum_{P\in S_{N}}\xi^{N_{P}}\int dr_{1}dr_{2}\cdots dr_{N}\langle P\left\{r\right\}|e^{-\beta \hat{H}}|\left\{r\right\}\rangle ,$$

where $\beta =1/k_{B}T$, $k_{B}$ is the Boltzmann constant, *T* is the system temperature, *P* represents the permutation operator, $S_{N}$ is the set of all elements in the permutation group concerning *N* fictitious identical particles. $S_{N}$ contains a total of N! permutation operators. ξ is a real parameter for fictitious identical particles, which signifies the weight factor after each particle permutation operation, $N_{P}$ is the minimum number of pair permutation operations required to restore the original order $\left(r_{1},\cdots ,r_{N}\right)$. It is evident from Equation (2) that when ξ=1, Equation (2) represents the partition function of identical bosons; whereas when ξ=−1, Equation (2) represents the partition function of identical fermions.

We will use the recursive formula for the parametrized partition function of fictitious identical particles in Ref. [36], which is based on the idea of recursive formula on the partition function of bosons and fermions by Hirshberg et al. [16,17]. The parametrized partition function is

(3) $$Z\left(\beta ,\xi \right)∼\int dR_{1}\cdots dR_{N}e^{-\beta U_{\xi }^{\left(N\right)}},$$

where

(4) $$U_{\xi }^{\left(N\right)}=-\frac{1}{\beta }\ln W_{\xi }^{\left(N\right)}+\frac{1}{P}\sum_{j=1}^{P}V\left(r_{1}^{j},\cdots ,r_{N}^{j}\right),$$

and

(5) $$W_{\xi }^{\left(N\right)}=\frac{1}{N}\sum_{k=1}^{N}\xi^{k-1}e^{-\beta E_{N}^{\left(k\right)}}W_{\xi }^{\left(N-k\right)},$$

and

(6) $$E_{N}^{\left(k\right)}=\frac{1}{2}m\omega_{P}^{2}\sum_{l=N-k+1}^{N}\sum_{j=1}^{P}{\left(r_{l}^{j+1}-r_{l}^{j}\right)}^{2},$$

where $\omega_{P}=\sqrt{P}/\left(\beta \hbar \right)$, *ℏ* is the reduced Planck constant.

Here, we adopt the method of massive Nosé–Hoover chains [44,45,46,47,48] to sample according to a canonical distribution. After obtaining a large amount of sampled data, the following energy estimator is used to estimate the energy

(7) $$\langle E\rangle =\frac{PdN}{2\beta }+\langle U\rangle +\langle V_{\xi }^{\left(N\right)}+\beta \frac{\partial V_{\xi }^{\left(N\right)}}{\beta }\rangle ,$$

where $W_{\xi }^{\left(N\right)}=\exp\left(-\beta V_{\xi }^{\left(N\right)}\right)$ and $U=\frac{1}{P}\sum_{j=1}^{P}V\left(r_{1}^{j},\cdots ,r_{N}^{j}\right)$. For the focus of this study, the density estimator can be provided by the following equation:

(8) $$n\left(r\right)=\langle \frac{1}{P}\sum_{l=1}^{N}\sum_{j=1}^{P}\delta \left(r_{l}^{j}-r\right)\rangle .$$

After achieving thermal equilibrium through molecular dynamics, a large amount of sampled data can be averaged using Equations (7) and (8) to obtain the system’s energy and density distributions.

### 2.2. The Extrapolation Method for Strongly Quantum Degenerate Fermi Gases and Its Application in Density Distribution

After obtaining data within the region of ξ≥0 through the above PIMD sampling simulation, we can use these data to simulate the physical properties of fermionic systems. To introduce the constant density semi-extrapolation method of this paper, we first briefly introduce two types of ξ-extrapolation methods for energy.

(1) The isothermal extrapolation method for energy. For the same temperature *T*, we can calculate the energy at different ξ. For example, in this paper, we will calculate the energy for 13 different ξ values in the range of ξ∈[0,1.2] with an interval of 0.1, starting from ξ=0. Then, we can use the following quadratic function

(9) $$E\left(\xi ,T\right)=a\left(T\right)+b\left(T\right)\xi +c\left(T\right)\xi^{2},$$

to fit these data, thereby obtaining the energy of the fermionic system at a certain temperature *T* when ξ=−1. When calculating the density distribution using isothermal extrapolation, the process is similar, just replace energy *E* with density distribution n(r).

The isothermal extrapolation method only works well for moderately high temperatures. Xiong and Xiong [37] have demonstrated through the example of an ideal gas that at extremely low temperatures or under high quantum degeneracy conditions, when ξ≥0, systems corresponding to different ξ values have similar energy values. This is mainly because at extremely low temperatures, whether they are bosons (ξ=1) or distinguishable particles (ξ=0), all particles tend to be in the ground state, while the energy of fermionic systems still significantly exceeds that of bosons. Therefore, at extremely low temperatures, E(ξ,T) in the region of −1≤ξ≤1 does not exhibit good analytical properties. This is the direct cause of the failure of the isothermal extrapolation method at extremely low temperatures.

(2) Constant energy semi-extrapolation method. In order to overcome the aforementioned difficulty, Xiong and Xiong proposed the constant energy semi-extrapolation method [37]. Under fixed energy, calculate the temperature *T* corresponding to different ξ values at the same energy, then determine the temperature *T* corresponding to the energy value of the fermionic system at ξ=−1 through the function $\xi_{E}\left(T\right)$. In this way, it is possible to bypass the so-called “black region” shown in the article [37]. Because even the ground-state energy of fermions is always higher than that of the “black region”, the constant energy semi-extrapolation does not use the data in the “black region”. The advantage of the constant energy semi-extrapolation method is not only to bypass the “black region”, but also to impose strong exact constraints on the form of the function $\xi_{E}\left(T\right)$ at T=0 when performing the constant energy semi-extrapolation method. For the sake of systematic representation, a simple derivation of this exact relationship is given below [37].

According to the Third Law of Thermodynamics, when T→0, S→0. Therefore, $\lim_{T\to 0}\frac{\partial S}{\partial T}$ can only take on a finite value. Thus, we have:

(10) $$\lim_{T\to 0}\frac{\partial E(\xi ,T)}{\partial T}=0.$$

According to the following formula regarding partial derivatives:

(11) $$\frac{\partial \xi_{E}\left(T\right)}{\partial T}=-\frac{\partial E(\xi ,T)/\partial T}{\partial E(\xi ,T)/\partial \xi },$$

then, we have

(12) $$\lim_{T\to 0}\frac{\partial \xi_{E}\left(T\right)}{\partial T}=0.$$

In the above derivation, we replaced the function ξ(E,T) with $\xi_{E}\left(T\right)$.

For a given energy, when we prepare a series of data $\left\{\xi_{1},T_{1}\right\},\cdots ,\left\{\xi_{n},T_{n}\right\}$ by performing ab initio simulations for T>0, we can apply Equation (12) as a strong exact condition when inferring and fitting towards T=0. Therefore, we consider this a kind of constant energy semi-extrapolation method. We cannot call it interpolation yet because we do not know the value of ξ at T=0 beforehand. After obtaining the function $\xi_{E}\left(T\right)$ by imposing the constraint given in Equation (12), we can determine the temperature of the fermionic system with energy *E* by setting ξ=−1.

Following the general idea of the constant energy semi-extrapolation method, as developed in the article [43], we now specifically consider the density distribution n(ξ,T,r). Here, r represents the coordinates (x,y). Considering the functional form between *E* and (ξ,T), we can also express n(ξ,T,r) as n(ξ,E(ξ,T),r). Therefore, we have:

(13) $$\frac{\partial n(\xi ,E(\xi ,T),r)}{\partial T}=\frac{\partial n(\xi ,E(\xi ,T),r)}{\partial E}\frac{\partial E(\xi ,T)}{\partial T}.$$

Using Equation (10), we immediately obtain:

(14) $$\lim_{T\to 0}\frac{\partial n(\xi ,T,r)}{\partial T}=0.$$

We can also employ a similar partial derivative formula as in Equation (11)

(15) $$\frac{\partial \xi_{n}\left(T,r\right)}{\partial T}=-\frac{\partial n(\xi ,T,r)/\partial T}{\partial n(\xi ,T,r)/\partial \xi },$$

therefore, we obtain

(16) $$\lim_{T\to 0}\frac{\partial \xi_{n}\left(T,r\right)}{\partial T}=0.$$

Here, we use the subscript *n* to represent the function ξ(n,T,r) equivalent to $\xi_{n}\left(T,r\right)$.

Though we have constraints for the energy in Equation (12) and for the density n(r) in Equation (16), we are not certain about the specific form of the functions $\xi_{E}\left(T\right)$ and $\xi_{n}\left(T\right)$. For brevity, we will use a general *p* to replace *E* and n(r) here. We will expand $\xi_{p}\left(T\right)$ in a Taylor series with respect to the temperature *T*, including higher-order terms of the function $\xi_{p}\left(T\right)$.

(17) $$\xi_{p}+\sum_{m\geq 2}d_{m}\left(p\right)\xi_{p}^{m}=a\left(p\right)+b\left(p\right)T^{2}+\sum_{k>2}c_{k}\left(p\right)T^{k}.$$

The coefficients a(p), b(p), $c_{k}\left(p\right)$, and $d_{m}\left(p\right)$ in Equation (17) are all unknown, but we can determine these coefficients through data fitting of the region where $\xi_{p}\geq 0$ obtained from PIMD/PIMC simulations. There is no first-order term with respect to temperature *T* in Equation (17), which is determined by the aforementioned constraint in Equation (16).

In subsequent calculations, the constant energy semi-extrapolation method mainly considers two forms of fitting functions, namely retaining up to the quadratic term in temperature ($T^{2}$ constant energy semi-extrapolation method),

(18) $$\xi_{p}+d\left(p\right)\xi_{p}^{2}=a\left(p\right)+b\left(p\right)T^{2},$$

and up to the cubic term in temperature ($T^{3}$ constant energy semi-extrapolation method).

(19) $$\xi_{p}+d\left(p\right)\xi_{p}^{2}=a\left(p\right)+b\left(p\right)T^{2}+c\left(p\right)T^{3}.$$

It is worth noting that Taylor expansion generally applies only to low-temperature conditions. When the temperature is relatively high, it may be appropriate to consider retaining higher-order terms in the Taylor expansion or to use the applicable isothermal extrapolation method at that time. The $T^{2}$ constant energy semi-extrapolation method is typically more applicable at low temperatures, while the $T^{3}$ constant energy semi-extrapolation method is better suited for high-temperature situations. The difference between these two extrapolation methods can be seen in Section 3. Of course, a promising direction for future research is to consider other more suitable function forms that satisfy specific constraint conditions for specific problems.

In this paper, the specific approach and technical details for conducting constant energy semi-extrapolation or constant density semi-extrapolation are as follows (taking $T^{3}$ constant energy semi-extrapolation as an example):

(1) Obtain the *p*-values (energy *E* or density n(r)) at 13 points in ξ∈[0,1.2] at different temperatures through PIMD simulations;
(2) Fit the above data to obtain the function $p_{\xi }\left(T\right)$ for *p* as a function of temperature at different ξ;
(3) Given a *p*-value, solve for the temperature *T* at different ξ where $p_{\xi }\left(T\right)=p$;
(4) Use the corresponding ξ, *T* as input data into Equation (19) for fitting, obtaining the corresponding coefficients a(p), b(p), c(p), and d(p);
(5) Set ξ=−1 in Equation (19) to determine the temperature *T* for the fermion system at the given value of *p*;
(6) Select a series of different *p*-values and repeat steps (3) to (5) to obtain the *p*-values of fermions at different temperatures.

In step (2), for the case of limited data, the choice of the fitting function and temperature range may impact the results. In this step, we typically use a polynomial for fitting. If the degree of the fitting function is too high and there is not much data calculated by PIMD, it may lead to an oscillating fitting curve, which is known as overfitting. Therefore, in this paper, we usually choose the highest degree of the fitting function to be 3 or 4 in order to avoid overfitting. In addition, during the fitting process, one should be cautious not to select an excessively wide temperature range. These technical details can help minimize the errors introduced during the fitting process.

During the semi-extrapolation, the monotonicity of the function $p_{\xi }\left(T\right)$ also plays a significant role in the results. If $p_{\xi }\left(T\right)$ is a monotonic function with respect to temperature *T*, the corresponding semi-extrapolation will be simpler. For example, if energy increases monotonically with temperature and decreases monotonically with ξ, this directly limits the choice of functions for semi-extrapolation, leading to more accurate results. However, for the density distribution n(ξ,T,r), its variation is not as simple for different ξ and *T*. For instance, in a quantum system in a harmonic trap, when *r* is small, where r=|r|, n(ξ,T,r) increases monotonically with ξ and decreases monotonically with temperature *T*; and when *r* is large, n(ξ,T,r) decreases monotonically with ξ and increases monotonically with temperature *T*; however, when *r* takes intermediate values, the behavior of n(ξ,T,r) becomes more complex, no longer a monotonic function of ξ or *T*. This complexity makes inferring the density distribution more challenging. Furthermore, in some cases, this extrapolation method may become infeasible. For certain values of n(r) for fermions, situations may arise where the value cannot be attained by n(r) of bosons at any temperature. These scenarios will be thoroughly analyzed in the next section (Section 3), and further solutions to this challenge will be provided.

## 3. Results

As an example of inferring the thermodynamic properties, especially the density distribution of a fermion system based on fictitious identical particles, we now consider 10 fictitious identical particles in a two-dimensional isotropic harmonic trap. We consider the presence of Coulomb-like interactions between the particles, and the potential term in the Hamiltonian operator Equation (1) takes the following form:

(20) $$V\left(r_{1},\cdots ,r_{N}\right)=\sum_{l=1}^{N}\frac{1}{2}m\omega^{2}r_{l}^{2}+\;\sum_{j=1}^{N}\sum_{k=j+1}^{N}\frac{\lambda }{|r_{j}-r_{k}|}.$$

In subsequent calculations, we always set ℏ=1, m=1, $k_{B}=1$, and ω=1. The parameter λ in Equation (20) represents the strength of the interaction.

Similar to previous studies [36,37], this work primarily focuses on method development rather than direct practical applications to specific fermion systems. To facilitate independent verification of the results presented in this paper, we only analyze ten fermions as an example. When developing methods to address fermion sign problems in the article, the study is limited to 4–6 fermions [17]. When simulating fermionic systems with a significantly larger number of fermions, we can progressively increase the number of particles for simulation and explore the finite-size effects within them [42].

Due to the need to simulate the energy and density distribution of fermion systems at low temperatures or high quantum degeneracy, even with as few as 10 fermions, the severe difficulty posed by the fermion sign problem prevents us from directly verifying the inferred results using the direct PIMD/PIMC method on supercomputers. Even for cases of weak or moderate quantum degeneracy, independently verifying using direct PIMD/PIMC strategies would require extensive simulations on supercomputers (on the order $O(10^{7})$ of CPU hours) (see [38,39,40,41,42]). For most researchers, this approach of independent verification through direct PIMD/PIMC simulations is often impractical. Therefore, this work adopts an alternative strategy.

We believe that in order to accurately simulate the thermodynamic properties of interacting fermionic gases, the ideal Fermi gas provides a good theoretical benchmark. Once we establish the applicability and range of the ξ extrapolation method for the ideal Fermi gas, we can hope to accurately infer the thermodynamic properties of repulsive interacting Fermi gases. In the case of repulsive interactions, the fermion sign problem is weaker compared to the ideal Fermi gas at the same temperature. At this point, isothermal extrapolation or constant energy (constant density) semi-extrapolation curves are simpler than in the non-interacting case (specifically, the curvature of the isothermal or constant energy curves is smaller), providing hope for accurately inferring the thermodynamic properties of repulsive interacting Fermi systems. It is worth noting that for attractive interactions, the fermion sign problem is more severe than in the ideal Fermi system, raising more subtle issues and additional difficulties regarding the success of the ξ-extrapolation method [15,43,49]. The attractive interaction is vital in the simulation of plasma physics and nuclear physics. To avoid path collapsing in these systems is a rather tricky problem, while this work does not address cases involving attractive interactions.

### 3.1. Non-Interacting Case

**Figure 1:**
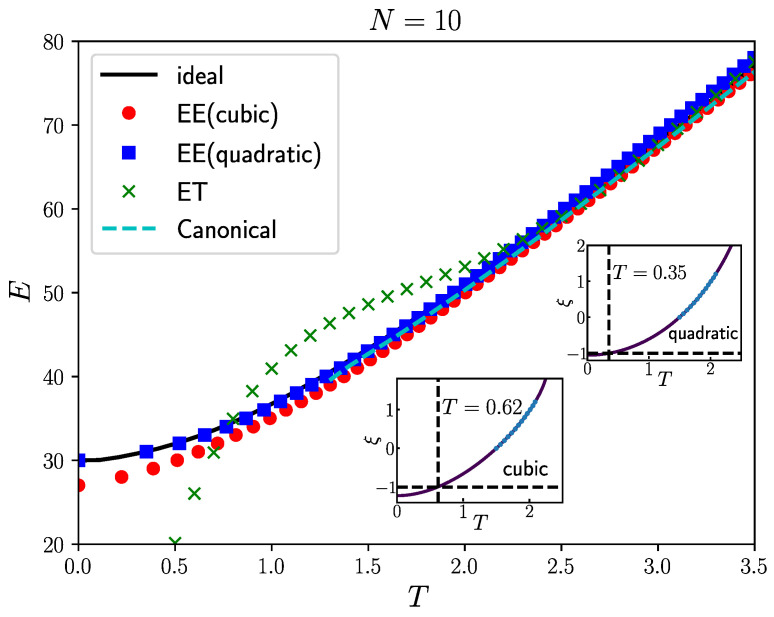
The energy of 10 non-interacting fermions in a two-dimensional harmonic trap at different temperatures. The black solid line represents the energy calculated based on the grand canonical distribution of the fermion system, the red circles represent the energy obtained from the $T^{3}$ constant energy semi-extrapolation using Equation (19), the blue squares represent the energy obtained from the $T^{2}$ constant energy semi-extrapolation using Equation (18), and the green crosses represent the energy obtained from the isothermal extrapolation. The cyan dashed line represents the result of the canonical ensemble for ideal fermions, using the recursion formula given in the article [22]. The cyan dashed line only extends to T=1.2 because the fermion sign problem still prevents reasonable results even for the ideal gas at lower temperatures. In the figure, “ideal” represents the grand canonical ensemble of ideal fermions, “EE (quadratic)” represents the $T^{2}$ constant energy semi-extrapolation, “EE (cubic)” represents the $T^{3}$ constant energy semi-extrapolation, “ET” represents the isothermal extrapolation, and “Canonical” represents the ideal canonical ensemble. The inset shows the fitted curve of the constant energy semi-extrapolation at energy E=31.0. We note that the applicability of the constant energy semi-extrapolation is evidently broader at low temperatures compared to the isothermal extrapolation.

**Figure 2:**
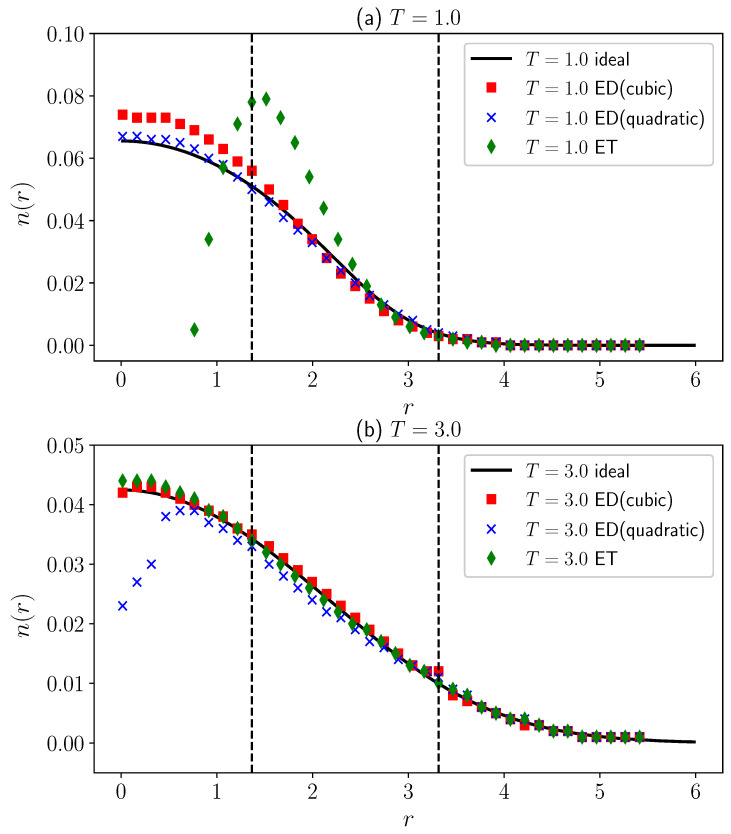
Density distribution of 10 non-interacting fermions in a two-dimensional harmonic trap at (**a**) T=1.0 and (**b**) T=3.0. The black solid line represents the result obtained from the grand canonical ensemble of fermions, while the red squares, blue crosses, and green diamonds are results obtained from ξ extrapolation methods. “ED(cubic)” denotes the $T^{3}$ constant density semi-extrapolation, “ED(quadratic)” denotes the $T^{2}$ constant density semi-extrapolation, and “ET” represents the isothermal extrapolation.

To verify the applicability of the extrapolation method based on fictitious identical particles, we first set λ=0, representing the case where there is no interaction between the particles. We studied the energy of non-interacting fermions (Figure 1) and their density distribution (Figure 2).

In the case of non-interacting fermions, for 10 fermions, the Fermi energy is $E_{F}=4.0$. The highest temperature considered in the graph is T=3.5, so the temperatures considered in the graph are all below the Fermi temperature, indicating a scenario where quantum degeneracy effects cannot be ignored. At relatively high temperatures (still considered low compared to the Fermi temperature) when T>2.5, the red circles (based on $T^{3}$ constant energy semi-extrapolation), blue squares (based on $T^{2}$ constant energy semi-extrapolation), green crosses (isothermal extrapolation), and black solid line (results from the grand canonical ensemble of ideal fermion system) in Figure 1 almost overlap, indicating that all three extrapolation methods are reliable at relatively high temperatures.

However, as the temperature decreases to T<2.0, the results from isothermal extrapolation start deviating significantly from the results of the grand canonical ensemble and canonical systems, with larger deviations as the temperature approaches zero. The results from $T^{3}$ constant energy semi-extrapolation show noticeable deviation from the grand canonical ensemble results only when T<1.0. Only the results from $T^{2}$ constant energy semi-extrapolation coincide with the results from the ideal grand canonical distribution when T<1.0. At low temperatures, the reason for the failure of isothermal extrapolation is that at low temperatures, all fictitious particles tend to accumulate in the same ground state when ξ>0. When ξ=−1, due to the Pauli exclusion principle, fermions cannot occupy the same quantum state. Therefore, at low temperatures, the analytical properties of the energy as a function of temperature exhibit significant differences on either side of ξ=0, leading to larger discrepancies between the energy obtained from isothermal extrapolation and the actual fermion energy at low temperatures.

Unlike isothermal extrapolation, constant energy semi-extrapolation can bypass the so-called “black region” and have additional exact conditions to adhere to, thereby providing an opportunity to accurately determine the energy of fermion systems at low temperatures. Nevertheless, when approaching zero temperature, $T^{3}$ constant energy semi-extrapolation may lead to deviation from the ideal values at low temperatures, likely due to overfitting, as evidenced by the agreement between the results from $T^{2}$ constant energy semi-extrapolation and the ideal results in the graph. Additionally, the inset displays the results of $T^{3}$ and $T^{2}$ constant energy semi-extrapolation at energy E=31.0. At the same energy, the temperature result from $T^{3}$ extrapolation is noticeably higher than that from $T^{2}$ extrapolation, indicating that at low temperatures, lower-order temperature terms should be used for extrapolation.

Figure 2 illustrates the density distribution obtained by the ξ-extrapolation method compared to the grand canonical ensemble. We use polar coordinates to simulate the density distribution n(r) and ensure that the distribution satisfies the normalization condition $\int_{0}^{+\infty }2\pi rn\left(r\right)dr=1$. When T=1.0 and r≲2.5, the quantum degeneracy effect is pronounced, showing significant differences in the properties of bosons and fermions. The isothermal extrapolation directly utilizes data from the quantum degenerate region for extrapolation, resulting in a substantial deviation from the ideal grand canonical distribution. Although the $T^{3}$ constant density semi-extrapolation aligns well with the ideal results for r≳1.365, as *r* decreases, the results gradually deviate from the black solid line due to overfitting at low temperatures. Notably, retaining only the $T^{2}$ term for the constant density semi-extrapolation shows minimal deviation from the ideal grand canonical distribution, as observed in Figure 2a. At T=3.0, both the $T^{3}$ constant density semi-extrapolation and isothermal extrapolation closely match the results of the grand canonical ensemble throughout the entire region. Conversely, the $T^{2}$ constant density semi-extrapolation exhibits a decrease in density values starting from r<1, contradicting the distribution pattern of an ideal gas. Results in Figure 2b indicate that the $T^{2}$ constant density semi-extrapolation is unsuitable for relatively high temperatures, requiring the inclusion of higher-order temperature terms for reasonable outcomes.

It should be noted that when calculating the density distribution of fermionic systems using the constant density semi-extrapolation method, we actually compute the density distribution only for r<1.365 and r>3.315. This limitation arises from the inconsistent monotonicity of density distributions at different positions with different temperatures, as discussed in the previous section (Section 2). Especially within the range 1.365<r<3.315, the density distribution of bosons cannot reach the values obtained by fermions at any temperature, making it impossible to define corresponding equal-density surfaces for extrapolation within this interval. However, we can circumvent this problem by choosing the appropriate range, which can be determined ad hoc according to the data we attained from the extrapolations. The density distribution in the range 1.365<r<3.315 in Figure 2 is obtained through interpolation. This interpolation method yields reasonably accurate results, compensating for the shortcomings of constant-density semi-extrapolation.

### 3.2. Interacting Case

Figure 1 and Figure 2 validate the feasibility of semi-extrapolations for equal energy and equal density in the case of an ideal Fermi system. Now, we consider examples of repulsive interactions, which have practical applications in quantum dot-related fields. The fermion sign problem with repulsive interactions is weaker compared to the non-interacting case. Therefore, after verifying and defining the applicable range for ideal gases, there is hope for reliable simulation of systems with repulsive interactions.

To validate the applicability of constant energy semi-extrapolation in the presence of interactions, we compared our results with those of Dornheim et al. [38]. Using a system of N=6 fictitious identical particles confined in a two-dimensional harmonic trap, Dornheim et al. observed that only under the conditions of weak quantum degeneracy could the energy obtained from isothermal extrapolation agree with results derived from the direct PIMC method. Conversely, when interactions are weak or absent, particularly under the condition of β=1, significant disparities arise between the energies from isothermal extrapolation and those obtained via the direct PIMC method.

**Figure 3:**
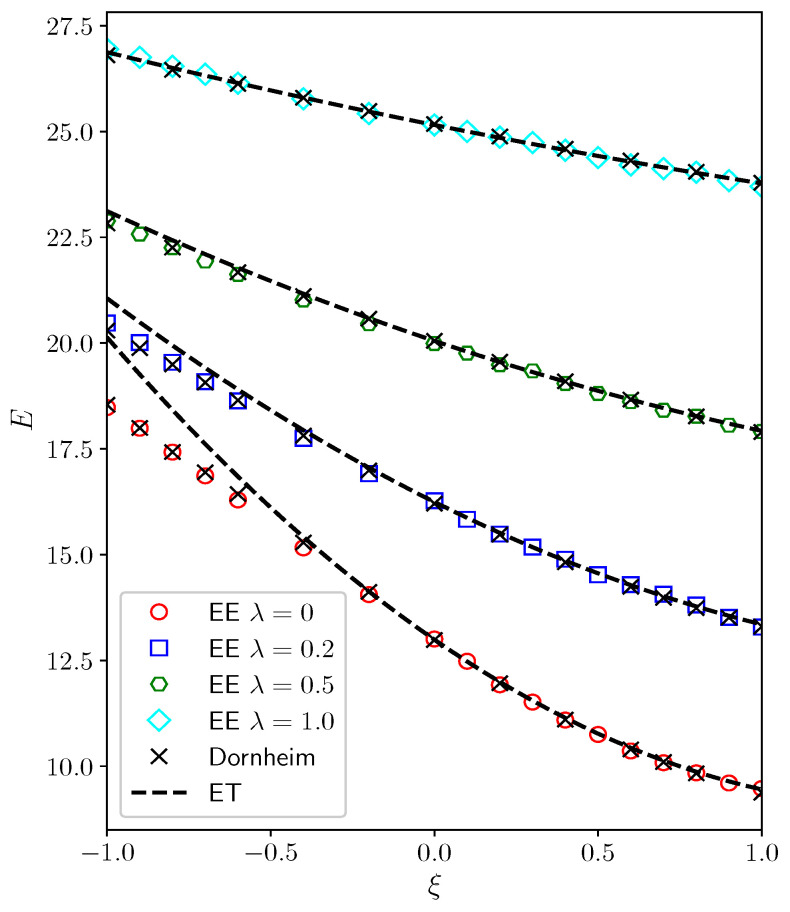
In a two-dimensional harmonic trap, the energy dependence on the parameter ξ of 6 fictitious identical particles under various interaction strengths is depicted. The energy profiles for λ=0,0.2,0.5,1.0 in the ξ≥0 region are represented by red circles, blue squares, green hexagons, and cyan diamonds, respectively, obtained from PIMD simulations, where results in the ξ<0 region are extrapolated using equal-energy extrapolation. The results using isothermal extrapolation are indicated by dashed lines, while crosses denote the results directly simulated by PIMC, as reported by Dornheim et al. [38].

Figure 3 illustrates the outcomes of constant energy semi-extrapolation (represented by hollow symbols) and isothermal extrapolation (indicated by dashed lines) at β=1. In the region where ξ≥0 and no fermion sign problem exists, results can be directly acquired from PIMD simulations. As depicted in the figure, the results from PIMD closely coincide with the PIMC results reported by Dornheim et al. [38], thus confirming the accuracy of our PIMD algorithm. Conversely, results in the ξ<0 region are obtained through both constant energy semi-extrapolation and isothermal extrapolation, with the latter’s outcomes represented by dashed lines in the figure. Notably, when λ=0.5 or 1.0, the energy derived from isothermal extrapolation closely matches the results obtained from the direct PIMC method (indicated by crosses). However, as λ decreases to 0 or 0.2, the discrepancy between the energies from isothermal extrapolation and the PIMC results increases with decreasing ξ. Conversely, across all depicted interaction strengths, the results obtained via constant energy semi-extrapolation (hollow symbols) closely align with those from the direct PIMC method. This disagreement between the dashed lines and crosses primarily stems from significant quantum degeneracy effects under β=1, particularly at interaction strengths λ=0 or 0.2, where the analytical properties of energy differ substantially between the ξ<0 and ξ≥0 regions. Direct isothermal extrapolation using data solely from this region results in deviations from the PIMC results. However, constant energy semi-extrapolation leverages data from temperatures higher than β=1 in the ξ≥0 region, effectively bypassing regions with disparate analytical properties and achieving better agreement with PIMC simulation results [38] in the ξ<0 region. This further validates the correctness of constant energy semi-extrapolation.

**Figure 4:**
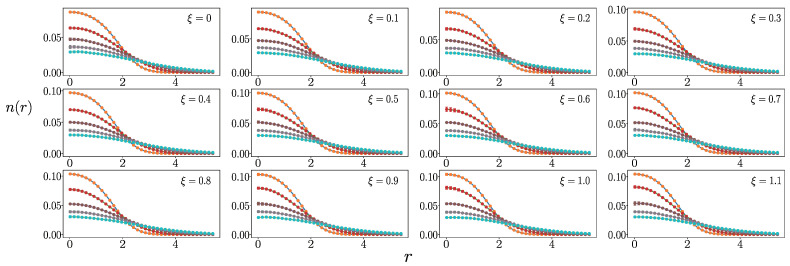
At λ=0.1, the figure presents the density distributions of 10 fictitious identical particles with different ξ values in a two-dimensional harmonic trap (12 different fictitious identical particles when ξ∈[0,1.1], obtained through PIMD simulations). Each of the 5 curves in every subplot corresponds to temperatures T=0.5, 1.5, 2.5, 3.5, 4.5. As the temperature increases, the density at r=0 gradually decreases, allowing us to infer the specific temperature values for each curve. For each density distribution, we conducted ten independent PIMD simulations and averaged the results.

For cases with interactions, in order to obtain the density distribution of a Fermi system using constant density semi-extrapolations, we still need to simulate the density distribution of fictitious identical particles at different temperatures for different values of ξ≥0 through PIMD. In Figure 4, we present partial density distribution results from PIMD simulations. It can be observed that the fluctuations in the density distribution results from PIMD simulations are very small, thus aiding in conducting accurate constant density semi-extrapolations.

**Figure 5:**
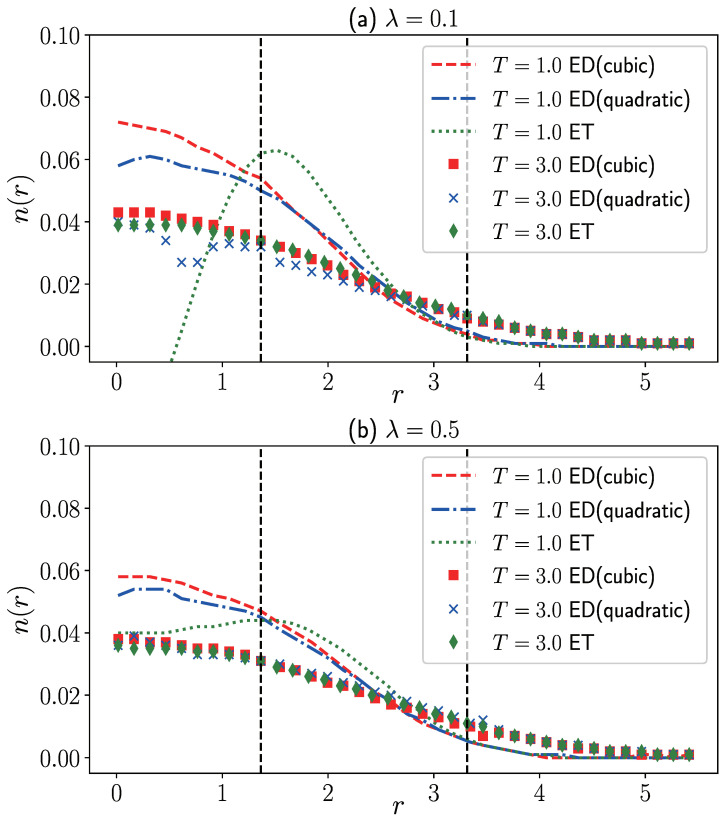
The density distribution of 10 fermions in a two-dimensional harmonic trap under different interaction strengths using constant density semi-extrapolations and isothermal extrapolation. Initially, identical particle density distributions within ξ∈[0,1.2] are obtained through PIMD simulations. Subsequently, the fermion system’s density distributions at various interaction strengths are derived using constant-density semi-extrapolation and isothermal extrapolation methods. Here, “ED(cubic)” denotes $T^{3}$ constant density semi-extrapolation, “ED(quadratic)” represents $T^{2}$ constant density semi-extrapolation, and “ET” indicates isothermal extrapolation.

Based on the density distribution at different temperatures and different values of ξ≥0, we can infer the density distribution of the Fermi system at different temperatures through isothermal extrapolation and constant density semi-extrapolations. Figure 5a presents the density distribution for weak interactions (λ=0.1). It is noted that at T=1.0, the density distribution from isothermal extrapolation (blue dashed line) exhibits significant physical inaccuracies with spurious peaks for r<2.5. However, for r>2.5, the density distribution from isothermal extrapolation matches the results from constant density semi-extrapolation. This phenomenon has also been observed in Dornheim’s study on the isothermal extrapolation of density distribution [38]. For the fermion system confined in a harmonic trap, at the same temperature, the quantum degeneracy is stronger closer to the center and weaker towards the edge. This position-dependent quantum degeneracy determines the applicability range of isothermal extrapolation, which depends on the spatial region. Fortunately, at T=1.0, from Figure 5, we observe that both $T^{3}$ and $T^{2}$ constant density semi-extrapolation provide reasonable density distributions throughout the entire region. However, deviations occur for r≲1.365, and these discrepancies increase as *r* decreases. The correctness of both results cannot be determined at present, and additional supporting results from other methods are needed. It is worth noting that the curve for $T^{2}$ constant density semi-extrapolation is not very smooth at small *r*, which may be primarily due to significant density fluctuations as *r* approaches 0. At T=3.0, the quantum degeneracy weakens, thus expanding the applicability range of isothermal extrapolation. It is observed that at T=3.0, isothermal extrapolation and $T^{3}$ constant density semi-extrapolation exhibit relatively good consistency throughout the entire region. However, for $T^{2}$ constant density semi-extrapolation, a physically unreasonable minimum occurs at r≈0.6, indicating the need to retain higher-order temperature terms at relatively high temperatures.

In Figure 5b, we present the density distributions of isothermal extrapolation and constant density semi-extrapolations at λ=0.5. When the repulsive interactions are strengthened, we expect the applicability range of isothermal extrapolation to increase at the same temperature. At T=1.0, we can observe this conclusion by comparing Figure 5a,b. In specific studies, compared to isothermal extrapolation, of course, constant-density semi-extrapolation can yield more reasonable results. However, in practical research, isothermal extrapolation still holds value in testing the reasonableness of constant-density semi-extrapolation through comparisons. This is also why we consistently use both methods of ξ-extrapolation for studying and comparing in this article. Additionally, the constant density semi-extrapolation results of $T^{2}$ and $T^{3}$ are closer at λ=0.5 compared to λ=0.1, indicating that as repulsive interactions strengthen, the dependence of fermionic system properties on the extrapolation function form is relatively small. At T=3.0, it can be seen that the results of the three extrapolation methods almost overlap. Strong interactions and relatively high temperatures weaken the quantum degeneracy effect of the system, making the analytical properties of the bosonic and fermionic regions closer, so regardless of the extrapolation method chosen, the results are relatively reasonable. Furthermore, similar to the ideal Fermi system, we cannot directly obtain the density distribution of the fermionic system at 1.365<r<3.315 through constant density semi-extrapolation. This limitation does not apply to isothermal extrapolation, which is suitable for all distance ranges. The reason is similar to the analysis of Figure 2; within this interval, the relationship between density values and temperature is not monotonic. In most cases, there are no equal-density surfaces, so it is also impossible to calculate the Fermi density distribution using constant-density semi-extrapolation. Therefore, the results at 1.365<r<3.315 in the figure are obtained through interpolation fitting.

**Figure 6:**
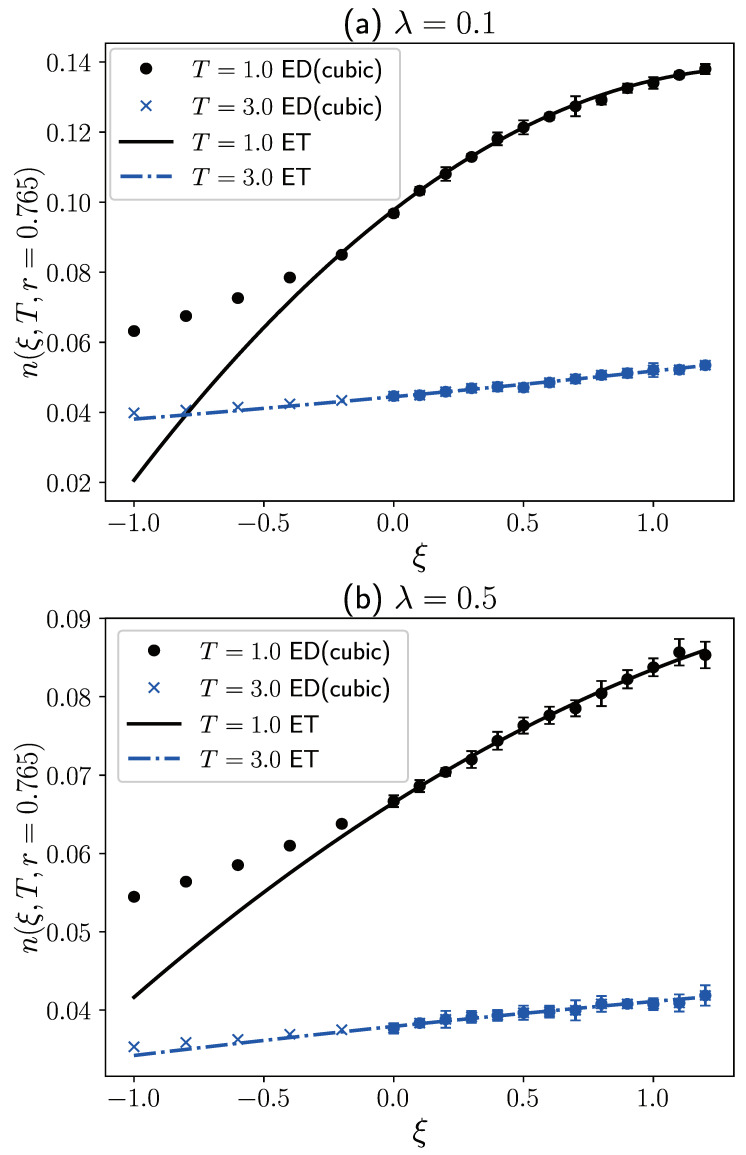
In a two-dimensional harmonic trap, the density distributions at r=0.765 of 10 fictitious identical particles under different interaction strengths and temperatures are extrapolated using $T^{3}$ constant-density semi-extrapolation and isothermal extrapolation. The results for ξ≥0 are obtained from PIMD simulations, while those for ξ<0 are obtained through $T^{3}$ constant-density semi-extrapolation or isothermal extrapolation. The error bars in the figure represent the standard deviation of the PIMD simulation results with different initial positions and velocities.

To further illustrate the difference between constant-density semi-extrapolation and isothermal extrapolation, Figure 6 provides extrapolation results of the density distribution at r=0.765 under different interaction strengths and temperatures. At T=1.0, in both λ=0.1 and λ=0.5 cases, isothermal extrapolation incorrectly estimates the density distribution in the ξ<0 region. In contrast, $T^{3}$ constant-density semi-extrapolation can exhibit the inflection point mentioned in the work of Dornheim et al. [38], making its results closer to real fermionic systems. At T=3.0, the results of isothermal extrapolation and $T^{3}$ constant-density semi-extrapolation are relatively close, mainly due to high temperatures suppressing quantum degeneracy.

### 3.3. Ab Initio Simulation of Entropy in Fermi Systems

In addition to being applicable to high quantum degeneracy, another advantage of $T^{3}$ constant-energy semi-extrapolation is the ability to obtain the energy E(T) of Fermi systems at different temperatures from zero temperature to finite temperature continuously. In comparison, isothermal extrapolation requires independent energy simulations in the bosonic region and ξ extrapolation at each temperature. To demonstrate this advantage of constant-energy semi-extrapolation, we use ab initio simulation of entropy as an example for analysis. Based on the differential relationship between entropy and energy, we have

(21) $$S\left(T\right)=\int_{0}^{T}\frac{dE}{T}.$$

**Figure 7:**
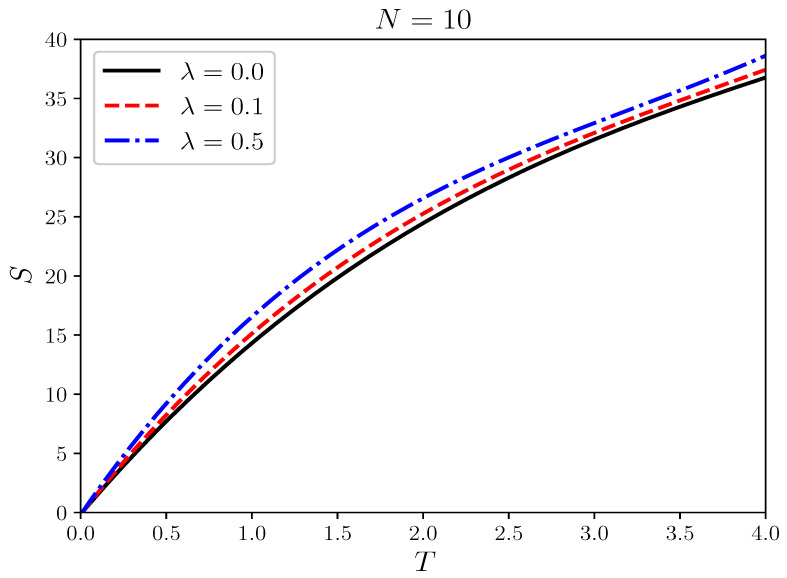
The variation of entropy with temperature for a 10-fermion system in a two-dimensional harmonic trap under different interaction strengths. The solid black line, dashed red line, and dotted blue line correspond to the entropy for λ=0, 0.1, 0.5, respectively.

Figure 7 illustrates the relationship between entropy and temperature for 10 fermions in a harmonic trap under different interactions. As the temperature increases, regardless of the interaction strength, the system’s disorder will increase, as shown in Figure 7, where the entropy increases with temperature for three interaction strengths. Moreover, a larger λ implies stronger repulsive interactions, leading to a more dispersed particle distribution and a lower probability of particles being in the ground state. Therefore, the system’s disorder increases, and correspondingly, the entropy increases as well. It is worth noting that the three curves in Figure 7 do not intersect even at near-zero temperatures, which is physically reasonable and supports the reliability of the constant energy semi-extrapolation. The reason for such stable results is partly because of the strong constraint at zero temperature in extrapolations, i.e., $\frac{\partial E}{\partial T}=0$.

## 4. Conclusions

In summary, in this work, we have extended the idea of constant energy semi-extrapolation to density distribution simulations and found promising and reliable results in cases of high quantum degeneracy. In contrast, isothermal extrapolation fails to produce reasonable density distributions under conditions of high quantum degeneracy. We compared the energy and density distribution results of fermionic systems obtained using the fictitious identical particle-based constant energy (or constant density) semi-extrapolation and isothermal extrapolation methods with ideal fermi systems and parts of the results in interacting fermi systems. Overall, constant energy (or constant density) semi-extrapolations based on $T^{3}$ and $T^{2}$ are superior to isothermal extrapolation, especially in cases of weak repulsive interactions and low temperatures. At high temperatures (around T=3.0), $T^{3}$ constant energy (constant density) semi-extrapolation is better than $T^{2}$, whereas, at low temperatures (around T=1.0), $T^{2}$ constant energy (constant density) semi-extrapolation results are more in line with expectations. However, a constant density distribution extrapolation may not accurately infer the fermion density distribution when the distance *r* is in the middle range, as the density distribution in this interval is not strictly monotonic with temperature. Some density values in this range do not even have corresponding equal density surfaces, limiting the applicability of constant density distribution extrapolation in this interval. We partially address this issue through interpolation. Regarding this density distribution challenge, the extension of physical information neural networks to simulate density distribution, as discussed in the paper [49], is a promising avenue worth exploring for further addressing these challenges in the future. For neural networks, accumulating more data in the bosonic region during training helps the accuracy of density extrapolation. It can be easily extended to the calculations of other physical quantities. In this work, the crosscheck in interacting fermi systems is limited because of the lack of data in such systems. More verification in interacting systems should be included in future works. Another interesting point is the correlation effects in interacting Fermi systems, and we will study this problem in future works. Although the ξ extrapolation method has already been successfully applied to warm dense matter and liquid Helium systems, there are still new avenues for future exploration, like quantum chemistry, nuclear physics, and cold atoms.

## Data Availability

The raw data supporting the conclusions of this article will be made available by the authors on request.

## References

[1] Ceperley D.M., Binder K., Ciccotti G. (1996). Path Integral Monte Carlo Methods for Fermions. Monte Carlo and Molecular Dynamics of Condensed Matter Systems.

[2] Troyer M., Wiese U.J. (2005). Computational Complexity and Fundamental Limitations to Fermionic Quantum Monte Carlo Simulations. Phys. Rev. Lett..

[3] Dornheim T., Groth S., Bonitz M. (2018). The uniform electron gas at warm dense matter conditions. Phys. Rep..

[4] Alexandru A., Basar G., Bedaque P.F., Warrington N.C. (2022). Complex paths around the sign problem. Rev. Mod. Phys..

[5] Bonitz M., Vorberger J., Bethkenhagen M., Böhme M., Ceperley D., Filinov A., Gawne T., Graziani F., Gregori G., Hamann P. (2024). Toward First Principle Simulations of Dense Hydrogen. Phys. Plasmas.

[6] Martin R.M., Reining L., Ceperley D.M. (2016). Interacting Electrons: Theory and Computational Approaches.

[7] Feynman R.P., Albert R.H. (1965). Quantum Mechanics and Path Integrals.

[8] Tuckerman M.E. (2010). Statistical Mechanics: Theory and Molecular Simulation.

[9] Fosdick L.D., Jordan H.F. (1966). Path-integral calculation of the two-particle Slater sum for He^4^. Phys. Rev..

[10] Herman M.F., Bruskin E.J., Berne B.J. (1982). On path integral Monte Carlo simulations. J. Chem. Phys..

[11] Ceperley D.M. (1995). Path integrals in the theory of condensed helium. Rev. Mod. Phys..

[12] Boninsegni M., Prokof’ev N.V., Svistunov B.V. (2006). Worm Algorithm for Continuous-Space Path Integral Monte Carlo Simulations. Phys. Rev. Lett..

[13] Boninsegni M., Prokof’ev N.V., Svistunov B.V. (2006). Worm algorithm and diagrammatic Monte Carlo: A new approach to continuous-space path integral Monte Carlo simulations. Phys. Rev. E.

[14] Spada G., Giorgini S., Pilati S. (2022). Path-integral Monte Carlo worm algorithm for Bose systems with periodic boundary conditions. Condens. Matter.

[15] Morresi T., Garberoglio G. (2025). Normal liquid ^3^He studied by path-integral Monte Carlo with a parametrized partition function. Phys. Rev. B.

[16] Hirshberg B., Rizzi V., Parrinello M. (2019). Path integral molecular dynamics for bosons. Proc. Natl. Acad. Sci. USA.

[17] Hirshberg B., Invernizzi M., Parrinello M. (2020). Path Integral Molecular Dynamics for Fermions: Alleviating the Sign Problem with the Bogoliubov Inequality. J. Chem. Phys..

[18] Feldman Y.M.Y., Hirshberg B. (2023). Quadratic Scaling Bosonic Path Integral Molecular Dynamics. J. Chem. Phys..

[19] Myung C.W., Hirshberg B., Parrinello M. (2022). Prediction of a supersolid phase in high-pressure deuterium. Phys. Rev. Lett..

[20] Yu Y., Liu S., Xiong H., Xiong Y. (2022). Path integral molecular dynamics for thermodynamics and Green’s function of ultracold spinor bosons. J. Chem. Phys..

[21] Xiong Y., Xiong H. (2022). Path integral molecular dynamics simulations for Green’s function in a system of identical bosons. J. Chem. Phys..

[22] Xiong Y., Liu S., Xiong H. (2024). Quadratic scaling path integral molecular dynamics for fictitious identical particles and its application to fermion systems. Phys. Rev. E.

[23] Higer J., Feldman Y.M.Y., Hirshberg B. (2025). Periodic Boundary Conditions for Bosonic Path Integral Molecular Dynamics. arXiv.

[24] Ceperley D.M. (1991). Fermion nodes. J. Stat. Phys..

[25] Schoof T., Groth S., Vorberger J., Bonitz M. (2015). *Ab Initio* Thermodynamic Results for the Degenerate Electron Gas at Finite Temperature. Phys. Rev. Lett..

[26] Ceperley D.M. (1992). Path-integral calculations of normal liquid ^3^He. Phys. Rev. Lett..

[27] Militzer B., Pollock E.L., Ceperley D.M. (2019). Path integral Monte Carlo calculation of the momentum distribution of the homogeneous electron gas at finite temperature. High Energy Dens. Phys..

[28] Mak C.H., Egger R., Weber-Gottschick H. (1998). Multilevel blocking approach to the fermion sign problem in path-integral Monte Carlo simulations. Phys. Rev. Lett..

[29] Blunt N.S., Rogers T.W., Spencer J.S., Foulkes W.M. (2014). Density-matrix quantum Monte Carlo method. Phys. Rev. B.

[30] Schoof T., Bonitz M., Filinov A.V., Hochstuhl D., Dufty J.W. (2011). Configuration Path Integral Monte Carlo. Contrib. Plasma Phys..

[31] Groth S., Dornheim T., Sjostrom T., Malone F.D., Foulkes W.M.C., Bonitz M. (2017). *Ab initio* exchange-correlation free energy of the uniform electron gas at warm dense matter conditions. Phys. Rev. Lett..

[32] Carlson J., Gandolfi S., Schmidt K.E., Zhang S. (2011). Auxiliary-field quantum Monte Carlo method for strongly paired fermions. Phys. Rev. A.

[33] Qin M., Shi H., Zhang S. (2016). Benchmark study of the two-dimensional Hubbard model with auxiliary-field quantum Monte Carlo method. Phys. Rev. B.

[34] Prokof’ev N.V., Svistunov B.V. (2007). Bold diagrammatic Monte Carlo technique: When the sign problem is welcome. Phys. Rev. Lett..

[35] Hou P.C., Wang B.Z., Haule K., Deng Y., Chen K. (2022). Exchange-correlation effect in the charge response of a warm dense electron gas. Phys. Rev. B.

[36] Xiong Y., Xiong H. (2022). On the thermodynamic properties of fictitious identical particles and the application to fermion sign problem. J. Chem. Phys..

[37] Xiong Y., Xiong H. (2023). On the thermodynamics of fermions at any temperature based on parametrized partition function. Phys. Rev. E.

[38] Dornheim T., Tolias P., Groth S., Moldabekov Z.A., Vorberger J., Hirshberg B. (2023). Fermionic physics from *ab initio* path integral Monte Carlo simulations of fictitious identical particles. J. Chem. Phys..

[39] Dornheim T., Schwalbe S., Moldabekov Z.A., Vorberger J., Tolias P. (2024). *Ab initio* path integral Monte Carlo simulations of the uniform electron gas on large length scales. J. Phys. Chem. Lett..

[40] Dornheim T., Döppner T., Tolias P., Böhme M., Fletcher L., Gawne T., Graziani F., Kraus D., MacDonald M., Moldabekov Z. (2024). Unraveling electronic correlations in warm dense quantum plasmas. arXiv.

[41] Dornheim T., Schwalbe S., Böhme M.P., Moldabekov Z.A., Vorberger J., Tolias P. (2024). Ab initio path integral Monte Carlo simulations of warm dense two-component systems without fixed nodes: Structural properties. J. Chem. Phys..

[42] Dornheim T., Schwalbe S., Tolias P., Moldabekov M.P.B.Z.A., Vorberger J. (2024). *Ab initio* Density Response and Local Field Factor of Warm Dense Hydrogen. Matter Radiat. Extremes.

[43] Xiong Y., Xiong H. (2024). Ab initio simulation of the universal properties of unitary Fermi gas in a harmonic trap. arXiv.

[44] Nosé S. (1984). A molecular dynamics method for simulations in the canonical ensemble. Mol. Phys..

[45] Nosé S. (1984). A unified formulation of the constant temperature molecular dynamics methods. J. Chem. Phys..

[46] Hoover W.G. (1985). Canonical dynamics: Equilibrium phase-space distributions. Phys. Rev. A.

[47] Martyna G.J., Klein M.L., Tuckerman M. (1992). Nosé-Hoover chains: The canonical ensemble via continuous dynamics. J. Chem. Phys..

[48] Jang S., Voth G.A. (1997). Simple reversible molecular dynamics algorithms for Nosé-Hoover chain dynamics. J. Chem. Phys..

[49] Xiong Y., Xiong H. (2024). *Ab initio* simulations of the thermodynamic properties and phase transition of Fermi systems based on fictitious identical particles and physics-informed neural networks. arXiv.

